# Novel pulsed field ablation application method for small pulmonary veins using a pentaspline catheter: The “Jellyfish Method”

**DOI:** 10.1016/j.hrcr.2025.08.033

**Published:** 2025-09-04

**Authors:** Takashi Kanda, Hitoshi Minamiguchi, Yuki Shibuya, Takashige Sakio, Mikiko Matsumura, Osamu Iida

**Affiliations:** 1 Cardiovascular Division, Advanced Cardiac Rhythm Management Center, Osaka Keisatsu Hospital, Osaka, Japan; 2 Cardiovascular Division, Osaka Keisatsu Hospital, Osaka, Japan

**Keywords:** Pulsed field ablation, Pentaspline, Jellyfish method, Small pulmonary veins, Atrial fibrillation

**Key Teaching Points**

• The jellyfish method is a novel and effective troubleshooting technique for pentaspline pulsed field ablation (PFA) in small pulmonary veins (PVs). The method offers a practical solution for common impedance errors and unstable catheter contact that frequently occur in small PV anatomies, without requiring a change in the ablation platform.
• The jellyfish configuration achieves uniform and deep lesion formation through optimal electrode-tissue contact. This configuration, which aligns the catheter electrodes in a parallel direction to the PV course, leverages biophysical principles to enhance the electroporation effect, resulting in effective lesion formation that is crucial for durable PV isolation.
• A simple, reproducible method can address anatomic limitations in PFA. The jellyfish method demonstrates that a straightforward modification of a standard catheter configuration can overcome anatomic challenges, expanding the applicability of the pentaspline PFA system to a wider range of patients and PV anatomies.

## Introduction

Pulsed field ablation (PFA) is a rapidly expanding treatment for atrial fibrillation (AF), offering tissue-selective ablation with a low incidence of pulmonary vein (PV) stenosis. The Farapulse system (Boston Scientific Inc), a single-shot PFA platform, uses a pentaspline catheter for PV isolation.^1^, ^2^, ^3^, ^4^, ^5^, ^6^ Although conventional basket and flower configurations are standard and constrained configurations such as “Olive” are being explored for durable lesions, challenges persist in effectively ablating small PVs.^7^ In these anatomies, catheter insertion can be difficult, and close spline proximity often leads to impedance increases, causing system errors (eg, “301,” “303”) that interrupt energy delivery. This compromises procedural efficiency. We propose a novel PFA application method, the “Jellyfish method,” for the pentaspline catheter. This report describes its experimental validation and successful clinical application in challenging cases.

## Jellyfish method application

**Figure 1:**
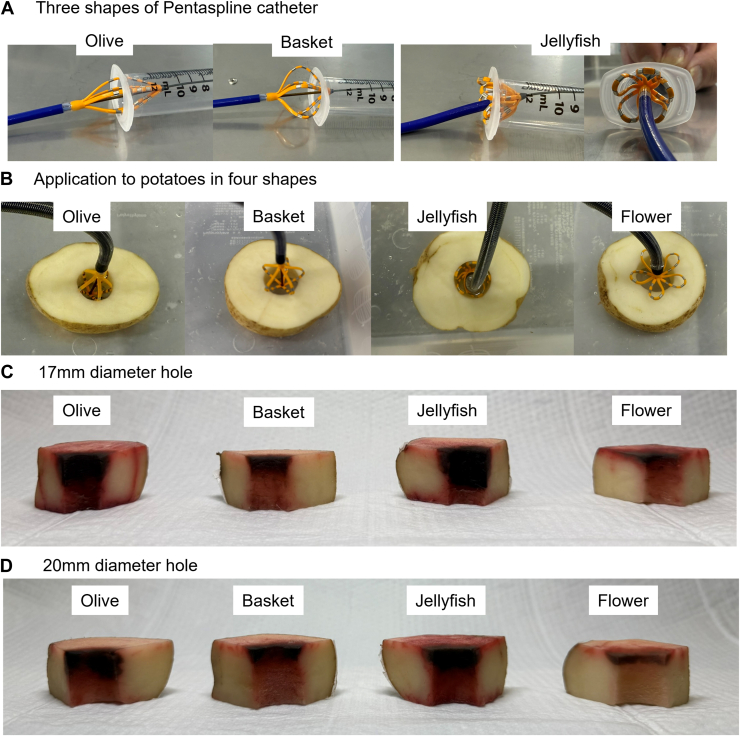
Pentaspline catheter configurations and in vitro potato model experiments. **A:** Images show the olive, basket, and jellyfish shapes. The rightmost image of the jellyfish shape demonstrates how adjacent petals are evenly distributed without overlapping even in a narrow lumen, which is crucial for uniform energy delivery. **B:** Setup for pulsed field ablation experiments using potato models. Potatoes were ablated with the pentaspline catheter in olive, basket, jellyfish, and flower configurations. Each application was performed twice at the same location without catheter rotation. **C:** Ablation effect in a 17 mm diameter potato hole. Images show cross-sections of the ablated potato after 24 hours. Lesion characteristics were evaluated by cutting the potato at 0°, 90°, 180°, and 270° from the site of deepest perpendicular depth. **D:** Ablation effect on a 20 mm diameter potato hole. Similar to panel C, this panel displays cross-sections of the ablated potato, evaluated by cutting into 4 pieces to assess lesion formation.

The jellyfish method is a novel technique for deploying the pentaspline catheter in situations where energy delivery with olive or basket configurations is difficult or impossible in small PVs. This technique enables easy formation of the jellyfish shape by maintaining the flower configuration, positioning the catheter centrally within the PV, and then advancing it while simultaneously rotating it. In particular, from the flower configuration, the sheath’s axis is aligned with the PV’s course. The catheter is then gently rotated and advanced into the PV (Supplemental Movie). This maneuver transforms the catheter into the jellyfish shape, characterized by the 5 petals (loops) inverting and splaying outward to ensure uniform contact with the PV wall (Figure 1A).

## In vitro potato model

To simulate small PVs, we prepared a well-established in vitro vegetal model using potato blocks, which are known for their reproducible composition and suitability for studying irreversible electroporation effects.^8^, ^9^, ^10^, ^11^, ^12^ We created 17 mm and 20 mm drilled holes, designed to mimic the anatomic characteristics of the small PVs. Ablation was performed in a 0.45% saline solution at 37°C. We tested flower, basket, olive, and jellyfish configurations, each with 2 sequences of energy delivery without rotation (Figure 1B). After ablation, lesion formation was assessed by treating the potato specimens with a triphenyl tetrazolium chloride solution. These effective ablation areas were then measured for lesion perpendicular depth and longitudinal depth from the PV entrance. The site of deepest lesion depth served as the reference point, from which evaluations were conducted at 0°, 90°, 180°, and 270°. The minimum depth was also recorded. In both 17 mm and 20 mm potato models, the jellyfish configuration consistently yielded sufficient, gap-minimal ablation effect in terms of penetration depth and distance from the entrance (Figure 1C and 1D). Its performance was particularly noteworthy in the 17 mm model (Figure 1C).

## Clinical case presentations

### Case 1

**Figure 2:**
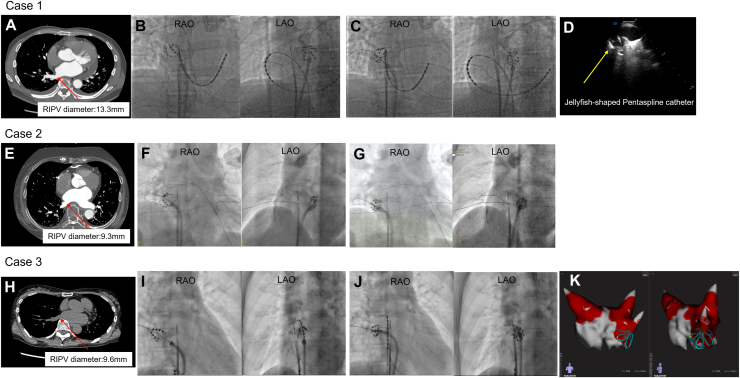
Clinical cases illustrating challenges in small pulmonary veins and resolution with the jellyfish method. **A:** Preprocedural CT scan of the RIPV in case 1, showing a small diameter of 13.3 mm. **B:** Fluoroscopic image during PFA in case 1, demonstrating the problematic basket configuration in the RIPV. Recurrent “301” errors occurred owing to close spline proximity. **C:** After conversion to the jellyfish configuration, successful, error-free energy delivery was achieved with uniformly spaced splines. **D:** Intracardiac echocardiography image in case 1, confirming excellent catheter-tissue contact of the jellyfish-shaped pentaspline catheter within the RIPV. **E:** Preprocedural CT scan of the RIPV in case 2, revealing a small diameter of 9.3 mm. **F:** Fluoroscopic image during PFA in case 2, showing the catheter forming a “cobra” shape during attempts with basket/olive configurations, leading to recurrent “301” errors and difficult catheter placement. **G:** Fluoroscopic image during PFA in case 2, demonstrating successful placement and energy delivery after transitioning to the jellyfish configuration. **H:** Preprocedural CT scan (noncontrast) of the RIPV in case 3, indicating a small diameter of 10 mm. **I:** Fluoroscopic image during PFA in case 3, illustrating the olive configuration failing owing to “303” errors from unstable contact. **J:** Fluoroscopic image during PFA in case 3, showing successful energy delivery after converting to the jellyfish configuration. **K:** FARAVIEW image from case 3, successfully displaying the jellyfish configuration, confirming its visibility on mapping systems. CT = computed tomography; PFA = pulsed field ablation; RIPV = right inferior pulmonary vein.

A 56-year-old man with paroxysmal AF underwent PFA. PV isolation was completed for the left superior, left inferior, and right superior PVs. For the right inferior PV (RIPV) (13.3 mm diameter on CT), basket configuration attempts caused recurrent “301” errors owing to close spline proximity (Figure 2A). After repositioning attempts failed, we used the jellyfish method. This allowed successful, error-free energy delivery with uniformly spaced splines (Figure 2B). Intracardiac echocardiography (ICE) also confirmed excellent catheter-tissue contact (Figure 2C).

### Case 2

An 81-year-old man with persistent AF had a challenging, very small RIPV isolation (9.3 mm diameter). Initial basket and olive configurations led to recurrent “301” errors. Furthermore, the catheter formed a “cobra” shape, making effective treatment difficult. Transitioning to the jellyfish method from the flower configuration achieved stable positioning and error-free energy delivery.

### Case 3

A 62-year-old woman with paroxysmal AF had a 10 mm RIPV. The conventional basket configuration caused intermittent “303” errors owing to unstable contact. The jellyfish method enabled more concentric and stable spline contact, allowing complete and uninterrupted PFA applications. In this case, FARAVIEW was used, and the jellyfish configuration was successfully displayed on FARAVIEW.

Across all 3 challenging clinical cases, the jellyfish method successfully resolved recurrent 301/303 errors or unstable contact encountered with conventional configurations, particularly in small-diameter PVs. Uniform spline distribution consistently allowed uninterrupted energy delivery (Figure 2C, 2G, and 2J). ICE confirmed excellent catheter-tissue contact with the PV wall (Figure 2D), and the jellyfish configuration was also successfully visualized on FARAVIEW (Figure 2K).

## Discussion

The pentaspline PFA catheter often faces impedance errors in small PVs owing to spline proximity. Our “Jellyfish method” offers a novel solution, validated experimentally and clinically. The PFA landscape is rapidly evolving with a variety of alternative platforms becoming available. However, given that the pentaspline catheter is widely used worldwide and supported by a rich body of evidence, refining techniques with this existing tool remains of significant importance. The anatomy of a patient’s PVs is not uniform, and there can be significant size differences even within a single patient. Therefore, our method presents a clinically useful approach for optimizing the performance of the pentaspline catheter, particularly in anatomically challenging cases. This method is particularly effective for small-diameter PVs where basket and olive configurations struggle to maintain sufficient distance between splines; in contrast, the jellyfish configuration, formed by inverting flower petals, ensures uniform spacing (Figure 1C and 1D), thereby minimizing 301/303 errors. Avoiding these errors is crucial not only for shortening procedural time but also for preventing potential damage to the console unit.

The importance of optimal electrode-tissue contact for durable lesion formation in PFA is well established. Furthermore, the inverted petals of the jellyfish configuration achieve excellent contact across more electrodes than conventional methods. This enhanced catheter-tissue contact leads to favorable, dense, and deep lesion formation (Table 1), with clinical ICE confirming excellent contact. Transforming the catheter into the jellyfish shape is also technically straightforward, achieved from the flower configuration by gentle rotation and advancement, which simplifies the procedure and potentially reduces procedural time. Although all 3 cases presented here involved the RIPV, our experience includes similar utility of the jellyfish method in other small PVs, such as the left inferior PV.

**Table 1:** Quantitative analysis of lesion characteristics from potato experiments

17 mm diameter hole		0° (maximum depth)	90°	180°	270°	Minimum depth
Perpendicular depth (mm)	Olive	4.0	2.5	2.0	1.7	1.4
	Basket	4.2	1.4	1.5	2.3	0.5
	Jellyfish	6.1	4.0	4.0	3.5	2.7
	Flower	11.6	6.3	2.2	6.8	1.0
Longitudinal depth (mm)	Olive	8.8	8.4	9.9	8.5	--
	Basket	4.6	3.9	4.2	2.0	--
	Jellyfish	10.1	13.9	12.4	9.4	--
	Flower	3.5	2.7	3.3	3.2	--

20 mm diameter hole		0° (maximum depth)	90°	180°	270°	Minimum depth
Perpendicular depth (mm)	Olive	3.0	0.8	2.7	2.3	0.5
	Basket	3.9	1.5	0.2	1.1	0
	Jellyfish	4.9	3.4	1.8	3.1	1.5
	Flower	4.7	3.2	1.2	0.8	0
Longitudinal depth (mm)	Olive	7.3	8.6	9.9	13.1	--
	Basket	4.4	3.1	0.1	3.1	--
	Jellyfish	4.9	5.4	6.1	9.8	--
	Flower	3.1	3.5	2.4	1.8	--

This table presents the perpendicular and longitudinal lesion depths in potato models with 17 mm and 20 mm diameter holes, comparing olive, basket, jellyfish, and flower configurations.

In the 17 mm diameter hole model, a comparison among olive, basket, and jellyfish configurations for perpendicular depth revealed that both olive and jellyfish created deep lesions. Notably, the jellyfish configuration also achieved a significantly greater minimum depth, indicating fewer gaps even without catheter rotation. For longitudinal depth, both olive and jellyfish configurations consistently created deep lesions. The flower configuration, being larger relative to the 17 mm hole, resulted in a wider perpendicular depth.

In the 20 mm diameter hole model, olive and jellyfish configurations again demonstrated superior perpendicular depth compared with basket. Although jellyfish achieved a greater minimum depth, the overall depths were shallower than those of the 17 mm model. Longitudinal depth similarly showed deep lesion formation with both olive and jellyfish configurations, consistent with the 17 mm model. For the flower configuration, the perpendicular depth was comparatively smaller in the 20 mm model than in the 17 mm model.

The pentaspline catheter is known for its ability to generate electric fields in 2 distinct vectors—a transverse field with the flower configuration and a longitudinal field with the basket configuration—thereby facilitating durable lesion formation by targeting myocardial fibers with varying orientations.^13^ Given the complex anatomy of the PV/left atrium, which is composed of multiple myocardial bundles arranged in diverse orientations, applying an electric field parallel to the tissue fibers results in a greater electroporation effect. The jellyfish method, similar to the basket and olive configurations, aligns its electrodes (except the fourth electrode) in parallel to the PVs. This biophysically rational alignment allows for effective electric field delivery deep within the PVs, making the jellyfish method a compelling alternative when basket or olive configurations are challenging for smaller PVs. This complementary electric field may enhance overall PFA efficacy when the basket configuration is difficult to achieve.

## Conclusion

The “jellyfish method” is a novel and promising PFA application technique, particularly beneficial for small PVs. Our study demonstrated its efficacy in achieving effective and uniform lesions in an in vitro vegetal model and successfully resolving energy delivery challenges in clinical cases. This innovative approach can expand the capabilities of the pentaspline PFA system.

## Disclosures

The authors have no conflicts of interest to disclose. The other authors have no conflicts of interest to disclose.
