# Supplementary material for: Novel pulsed field ablation application method for small pulmonary veins using a pentaspline catheter: The “Jellyfish Method”
Source: HeartRhythm Case Rep. 2025 Sep 4;11(12):1278–82. doi: 10.1016/j.hrcr.2025.08.033 (PMC12805270; doi:10.1016/j.hrcr.2025.08.033)
Supplement: Supplementary Video Legend [file mmc2.docx]

**Supplementary Movie.** Transformation to Jellyfish configuration and deployment in a small right inferior pulmonary vein

This supplemental movie demonstrates the simple technique to transform the pentaspline catheter from a Flower configuration to the Jellyfish shape. Following energy delivery in the Flower configuration within the right inferior pulmonary vein, the catheter is gently advanced while slowly rotating it. This maneuver easily transforms the catheter into the Jellyfish configuration, allowing for deployment within the narrow RIPV.
